# Seroprevalence and molecular detection of foot and mouth disease virus in cattle in selected districts of Wolaita Zone, Southern Ethiopia

**DOI:** 10.1038/s41598-024-57404-4

**Published:** 2024-04-04

**Authors:** Tamenech Bandaw, Haben Fesseha Gebremeskel, Ayelech Muluneh, Tilaye Shibiru Mengistu, Isayas Asefa Kebede

**Affiliations:** 1 School of Veterinary Medicine, Wolaita Sodo University, P. O. Box 138, Wolaita Sodo, Ethiopia; 2 Animal Health Institute, P. O. Box 04, Sebeta, Ethiopia; 3 School of Veterinary Medicine, Ambo University, P. O. Box 19, Guder, Ethiopia

**Keywords:** Cattle, FMD virus, Molecular detection, SAT-2, Seroprevalence, Wolaita zone, Infectious-disease diagnostics, Infectious diseases, Viral infection

## Abstract

Foot and mouth disease (FMD) is a highly contagious, endemic, and acute viral cattle ailment that causes major economic damage in Ethiopia. Although several serotypes of the FMD virus have been detected in Ethiopia, there is no documented information about the disease's current serostatus and serotypes circulating in the Wolaita zone. Thus, from March to December 2022, a cross-sectional study was conducted to evaluate FMDV seroprevalence, molecular detection, and serotype identification in three Wolaita Zone sites. A multistage sample procedure was used to choose three peasant associations from each study region, namely Wolaita Sodo, Offa district, and Boloso sore district. A systematic random sampling technique was employed to pick 384 cattle from the population for the seroprevalence research, and 10 epithelial tissue samples were purposefully taken from outbreak individuals for molecular detection of FMDV. The sera were examined using 3ABC FMD NSP Competition ELISA to find antibodies against FMDV non-structural proteins, whereas epithelial tissue samples were analyzed for molecular detection using real-time RT-PCR, and sandwich ELISA was used to determine the circulating serotypes. A multivariable logistic regression model was used to evaluate the associated risk variables. The total seroprevalence of FMD in cattle was 46.88% (95% CI 41.86–51.88), with Wolaita Sodo Town having the highest seroprevalence (63.28%). As a consequence, multivariable logistic regression analysis revealed that animal age, herd size, and interaction with wildlife were all substantially related to FMD seroprevalence (p < 0.05). During molecular detection, only SAT-2 serotypes were found in 10 tissue samples. Thus, investigating FMD outbreaks and identifying serotypes and risk factors for seropositivity are critical steps in developing effective control and prevention strategies based on the kind of circulating serotype. Moreover, further research for animal species other than cattle was encouraged.

## Introduction

Animal diseases reduce cattle output and productivity by 50 to 60% per year, according to estimations by Ganeshkumar^1^, MoA, and ILRI^2^, among other sources. One of the animal ailments restricting industrial productivity is foot-and-mouth disease (FMD). It is a contagious, worldwide, and economically damaging viral disease that affects both domestic and wild animals with cloven hooves^3,4^. It has resulted in trade embargoes for animals and livestock products, and it is regarded to be an issue for livestock productivity and output^5^.

FMDV, the FMD-causing virus, is a member of the Picornaviridae family and the Aphthovirus genus. Asia 1, O, A, C, and the South African Territories (SAT) SAT 1, SAT 2, and SAT 3 are among the seven serotypes. Throughout history, the majority of the world's areas have had comprehensive records of one or more serotypes^6^. It is distinguished by vesicular eruptions in the mouth, foot, and udder, which are linked to fever, lameness, salivation, and anorexia^7^.

The FMD virus can be spread directly, mechanically through infected host-to-host contact^8^, or indirectly through contact with an environment and objects contaminated with FMDV-infected secretions and excretions, such as clothing, shoes, vehicles, and veterinary instruments^9,11^. Furthermore, unregulated international movements of ill cattle and their products exacerbate disease propagation^10^. FMD is the most contagious disease that affects animals with cloven hooves, according to Larska et al.^11^, and it may cause significant economic losses in sensitive species such as cattle, sheep, horses, pigs, dogs, goats, and water buffalo.

The disease is a global issue that has affected almost every country throughout the years. It is most common in parts of South America, Asia, Africa, and the Middle East^7^. Ethiopia is one of the majority African countries where FMD is believed to be prevalent. The disease is commonly described as the cattle disease with the biggest economic impact in many industrialized and developing countries^12^.

Despite the Ethiopian government's stated aim to improve disease conditions and promote meat and live animal exports, there is no formal policy in place to control FMD by vaccination and/or movement restriction^13^. The frequency of uncertified free animal movement and a lack of vaccination practices (quality, coverage, and timeliness) are two variables that contribute to the spread of FMD along the cattle market chain^14^.

The government provides vaccination services to farmers for other transboundary livestock diseases such as Peste des petits ruminants, lumpy skin disease, contagious bovine pleuropneumonia, African horse sickness, and sheep and goat pox, as well as the FMD^15^. However, except for a limited number of market-oriented producers in metropolitan and peri-urban regions, the vast majority of farmers do not immunize their herds against FMD. Farmers may be unwilling to use the vaccine due to a lack of market availability and the high cost of vaccinations^15^. Furthermore, this makes the use of immunization to manage FMD difficult for developing countries with limited resources, such as Ethiopia. Although FMD is common in Ethiopia, vaccines to prevent the disease are infrequently administered^13^.

Several factors, including inadequate disease surveillance, a lack of FMDV molecular characterization, difficulties implementing vaccination programs, an inadequate understanding of the origin of infection, the emergence of new virus topotypes and lineages, low levels of technical capability and biosecurity at the national level, limited farmer knowledge of FMD disease recognition, and a failure to timely report outbreaks, make it difficult to successfully control the disease^16^. Multiple FMD outbreaks have been documented in several parts of Ethiopia among cattle kept under diverse management practices and giving milk, meat, and other types of revenue to farmers in particular and the nation as a whole^17^.

Numerous studies on the seroprevalence, serotype identification, and related risk factors of FMD in cattle have been conducted in Ethiopia due to continuing outbreaks of the disease among cattle; yet, the study area lacks knowledge on these issues. Even though there have been several FMDV outbreaks in the Wolaita zone, there isn't enough information, not even a single documented case, to comprehend the illness's serostatus, the serotypes that are widespread in the zone as a whole, and the study location in particular. Moreover, investigating the circulating serotype in the current study area will tremendously contribute to mitigating (manufacturing-related vaccine) the disease impact on the livestock sector. Thus, the primary objectives of the study are to identify the serotype circulating in cattle in the Wolaita zone study districts, quantify seroprevalence, molecularly detect foot and mouth disease virus, and identify risk factors.

## Materials and methods

### Study area

**Figure 1:**
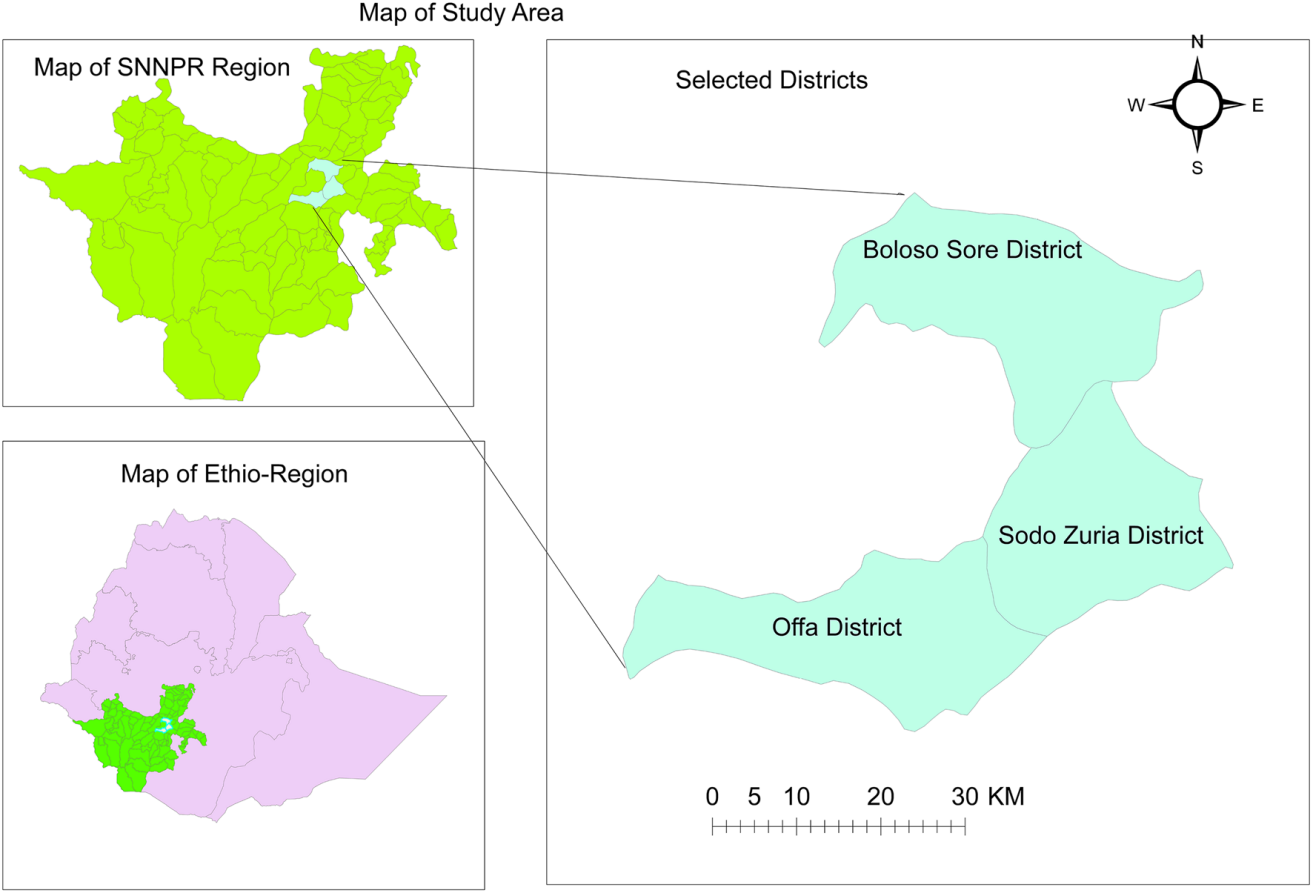
Map of the study sites (ArcGIS Software, 2024).

The study was conducted in three districts of southern Ethiopia's Wolaita Zone, including Offa District, Boloso Sore District, and Wolaita Sodo Town (Fig. 1). The research districts were chosen because they had a history of FMD outbreaks, a much greater cattle population, and a high amount of livestock exchanges via marketing with adjoining zones.

Wolaita Sodo Town is the first research site identified in Wolaita Zone. It is situated at 6°54′N 37°45′E and has an elevation of 1,600 to 2,100 m.a.s.l. The climate is subtropical highland, with rainy summers and dry winters. The area has a bimodal rainfall pattern from March to October. The average annual rainfall has been 1014 mm. The average yearly temperature is 19.9 °C, with monthly temperatures ranging from 17.7 °C in July to 22.1 °C in February and March.

Offa is a district located 29 km from Wolaita Sodo, the capital city of Wolaita. The climate of Offa Woreda is separated into three zones: Highland, Midland, and Lowland. It's at 37° 29′ 59.99" E and 6° 44′ 59.99" N, with an elevation range of 1200–2600 m.a.s.l. The annual rainfall in the area is between 800 and 1400 mm, while the temperature swings between 14 and 28 °C.

The third study location is Boloso Sore, which is bounded on the south by Sodo Zuria and Damot Sore, on the west by Boloso Bombe, on the northeast by the Kembata Tembaro Zone, on the east by Damot Pulasa, and on the southeast by Damot Gale. Areka serves as the administrative center. The area is around 300 km southwest of Addis Ababa, the capital. This settlement is located at 7° 4′ N, 37° 42′ E, and 1774 m.a.s.l.

### Study animals

Cattle of all ages were researched, including young (< 2 years), adult (2–4 years), and old (> 4 years), both sexes, and all breeds, in various husbandry and grazing systems (free, indoor, and mixed). In addition, following an outbreak of the disease, cattle in the district with characteristic FMD lesions were included for FMDV genetic detection and serotype identification. Small (< 10 cattle), medium (10–30 animals), and big (30 cattle) herds were randomly selected.

FMD is suspected when an animal exhibits excessive salivation and/or lameness. In addition, animals that had contact with the infected animal or had just been imported from the epidemic zone were tested. Furthermore, the oral cavity and hooves of the animals were examined for the presence of intact/ruptured vesicles, erosions, and ulcers. Cattle that had received the FMD vaccination during the preceding 6 months, on the other hand, were not sampled. Besides, the history of contact with the wildlife (buffalo) was recorded.

### Study design and sampling technique

Cross-sectional research was done in selected districts of the Wolaita zone from March to December 2022 to quantify FMD seroprevalence, detect FMDV serotypes, and analyze potential risk factors. Using a multistage sample technique, three kebeles (Peasant Associations, PAs) were selected from each research region: Wolaita Sodo, Offa district, and Boloso sore district. A systematic random sampling approach was used to select 384 cattle from the community for the seroprevalence investigation, and 10 calves were particularly selected from FMDV epidemic cases for molecular detection. The districts in this study were selected based on transportation accessibility, quick laboratory availability, historical disease incidence, and cattle population density.

### Sample size determination

The sample size for the seroprevalence study was estimated using Thrusfield's^18^ method. Because no prior research had been conducted in any section of the Wolaita zone, the sample size was estimated using a 50% anticipated prevalence (Pexp) with a 95% confidence interval (Z) and a 5% desired precision (d).

$$\begin{aligned} & {\text{n}} = \frac{{{{\text{Z}}^{2}}{\text{*Pexp*}}\left( {{1} - {\text{Pexp}}} \right)}}{{{{\text{d}}^{2}}}} \\ & {\text{n}} = \frac{{{1}.{9}{{6}^{2}}*0.{5*}\left( {{1} - 0.{5}} \right)}}{{{{\left( {0.0{5}} \right)}^{2}}}} = { 384} \\ \end{aligned}$$

The seroprevalence study comprised 384 cattle samples. In addition, during the study period, 10 cattle from epidemic cases in the study region were collected for molecular detection and serotype identification.

### Sample collection, transportation, and laboratory techniques

#### Serological test

For the seroprevalence investigation, approximately 10 ml of blood was aseptically taken from the jugular vein of cattle using vacutainer tubes. The blood was then transported to Wolaita Sodo Regional Laboratory to extract sera and preserve it until the serological test. First, the sera were allowed to clot overnight at room temperature; then it was transferred into sterile cryovials and finally, stored at − 20 °C until analysis. To do genetic analysis and identify the serotype, the sera were ultimately cold chain to the National Animal Health Diagnostic Investigation Centre (NAHDIC), located in Sebeta^19^.

##### Detection of antibodies against FMDV NSP by Competition ELISA

All sera were analyzed for antibodies using the ID Screen® FMD NSP Competition ELISA. This ID Screen® FMD NSP Competition ELISA kit is intended to identify specific antibodies in bovine serum against the FMDV NSP non-structural protein utilizing competition ELISA. While both infection and vaccination produce antibodies against structural antigens, only infected animals produce antibodies against the FMD virus's non-structural protein (NSP). When highly pure vaccinations are given, the FMDV NSP ELISA can be utilized as a DIVA test (Difference between Infected and Vaccinated Animals). The test distinguishes between samples from infected animals (presence of antibodies against NSP of FMD virus) and vaccinated animals (absence of antibodies against NSP of FMD virus)^20,21^.

The ELISA serology was performed at the NAHDIC laboratory using the ID Screen® FMD NSP Competition ELISA manufacturer's instructions and the protocol outlined in the OIE Manual of Diagnostic Tests and Vaccines for Terrestrial Animals^19^. In summary, each well received 50 µl of dilution buffer 18, 30 µl of the positive control to wells A1 and B1, 30 µl of the negative control to wells C1 and D1, and 30 µl of each sample to be examined to the other wells. 2 h at 37 °C. The wells were then emptied and cleaned five times with 200 µl of wash solution. Furthermore, each well got 100 µl of diluted conjugate. For 30 min, the test plates were sealed and incubated at 21 °C. The plates were then washed five times with 200 µl of the wash solution before 100 µl of the chromogen (Tetra-Methyl Benzidine) substrate was dispensed to all wells and incubated for 15 min at 21 °C in a dark place before 100 µl of the stop solution were added to all wells to stop the reaction and gently mixed. An EL × 800 BioTEK ELISA reader was used to measure optical density (OD) at 450 nm^19^.

If the mean value of the negative control OD (ODNC) is more than 0.7 (ODNC > 0.7) and the mean value of the positive control OD (ODPC) is less than 30% of the ODNC, the test result is validated (OD_PC_/OD_NC_ ≤ 0.3).

After that, the competition percentage was computed for each sample using the formula S/N% = OD sample/ODNC × 100. Positive S/N% was defined as less than or equal to 50% (S/N% 50%), while negative S/N% was defined as larger than 50% (S/N% > 50%). The ELISA test is a simple, quick, sensitive, and specific method for identifying antibodies to FMD virus non-structural proteins (NSP) in bovine serum. The real prevalence was thought to be the same as the percentage prevalence^21^.

#### Molecular detection

##### Tissue sample collection and preparation

For molecular detection and serotype identification of FMDV, samples were collected from cattle with evident clinical signs of FMD, such as an oral lesion, a history of infection but a healing lesion, and any additional asymptomatic animals on the same farm or grazing with the symptomatic cattle. Epithelial tissue samples were extracted from active, recently ulcerated, or burst vesicles using forceps and scissors. One gram of epithelial tissue was obtained from the tongue, foot, and gums and put in a container with viral transport media. According to Awel et al.^22^, the samples were accurately labeled and sent to the NAHDIC (Sebeta, Ethiopia) by cold chain for molecular analysis.

The samples were molecularly analyzed using the techniques indicated in the OIE guideline^23^. In brief, the epithelial samples were taken from the transport media and a suspension was prepared by grinding the sample in sterile solutions with a small amount of tissue using a sterile pestle and mortar.

Before sampling, the animal was restrained, and sick animals were subjected to a thorough physical examination. Following the completion of these processes, the sampling area was cleaned with alcohol, and the hairs were removed with a sterile scalpel^24^.

Anesthesia procedures and the anesthetic agent employed an 18-gauge 3.8-cm needle was inserted perpendicular to the skin's surface. Once the skin has been pierced, insert a drop of local anesthetic solution into the needle's hub. The needle should then be progressively advanced until the anesthetic solution was drawn into the subcutis. Lidocaine was used as an anesthetic. As previously described by Anderson and Edmondson^25^, 2% Lidocaine HCl (0.2 mg/kg body weight) was infused into the subcutis before sampling.

##### Extraction of viral RNA

Viral RNA was recovered from clinically FMD-infected epithelial tissue by preparing a suspension using a Qiagen RNA extraction kit and following the manufacturer's instructions, as reported by Kafeero et al.^26^. 140 µl (l) of sample suspension were added to 560 l of prepared buffer AVL containing carrier RNA in a 1.5 ml microcentrifuge tube. After mixing for 15 s, the mixture was incubated at 25 °C for 10 min to lyse. To eliminate drips from the inside of the lid, the tubes were gently centrifuged. The sample was then bonded by adding 560 l of 70% ethanol and mixing by pulse vertexing for 15 s, followed by centrifugation to remove droplets from the inner lid. The solution was then transferred to the QIAMP Minispin (silica) column in a 2 ml collection tube and centrifuged for 1 min at 8000 rpm.

After discarding the filtrate, the column was placed in a fresh 2 ml collection tube. Any remaining solution was similarly processed to acquire a bigger quantity of viral RNA material. First, 500 l of buffer AW1 was rinsed and centrifuged at 8000 rpm for 1 min. After discarding the filtrate, the column was placed in a fresh 2 ml collection tube. The column was then filled with 500 l of AW2 buffer and centrifuged at 14,000 rpm for 3 min. The column was then filled with 60 l of Buffer AVE and incubated at room temperature for one minute before being centrifuged at 8000 rpm for one minute. Finally, viral RNA was extracted. Extracted viral RNA was stored at + 4 °C until use or at 80 °C until further processing for real-time reverse transcription-polymerase chain reaction (rtRT-PCR)^27^.

##### Detection of FMDV by real-time RT-PCR

Extracted RNA samples were screened for FMDV presence using reverse transcription polymerase chain reaction (RT-PCR) with specific primers set FMDV7-forward (FMDV7F) and FMDV7-reverse (FMDV7R), as described by Dubie and Amare^28^.

A one-step rtRT-PCR assay was employed for FMDV detection. Real-time RT-PCR had a sensitivity similar to virus isolation, and automated methods boosted the sample throughout^29^. FMDV RNA reverse transcription and reverse-transcribed RNA PCR amplification were carried out utilizing automated one-step real-time RT-PCR, which recognizes the 3D RNA polymerase encoding gene^30^. The 3D non-structural protein viral RNA-dependent RNA polymerase is in charge of RNA replication and is highly conserved (94–99% similarity)^31^.

In the master mix reaction components, the forwarding primer (FMDV 3DF) 5-ACT GGG TTT TAC AAA CCT GTG A-3′, the reverse primer (FMDV 3DR) 5-GCG AGT CCT GCC ACG GA-3′, and the probe (FMDV Probe 3DP) 5-[6FAM] TCC TTT GCA CGC CGT GGG AC [TAM]-3′ were employed. The probe was labeled with 5-5-reporter dye, 6-carboxyfluorescein, and 3-quencher, tetramethyl rhodamine in a real-time RT-PCR technique to detect the 3Dpol gene sequence in all FMDV serotypes. For real-time RT-PCR, a superscript III/Platinum Taq one-step rRT-PCR kit was utilized. The master mix reaction components for one-step real-time RT-PCR were prepared using 12.5L of 2 × reaction mix, 1.5L of RNAse-free water, 2L of forward primer, 2L of reverse primer, 1.5L of TaqMan probe, and 0.5L of superscript ®III reverse transcription (RT), totaling 20L per sample for each PCR reaction per well per plate, including positive and negative control master mix. Pulse vertexing is then used to properly blend the mixture.

Each PCR plate included a 5 l extracted RNA template, for a total volume of 25 l. Before inserting the PCR plate into the thermal cycler machine slots and adjusting it according to the directions of the QIAgen one-step RT-PCR kit, it was sealed with adhesive tape. Initial denaturation at 95 °C for 10 min was followed by denaturation at 95 °C for 15 s, annealing at 60 °C for 1 min, and extension at 72 °C for 30 s. Amplifications were completed in 50 cycles for each run^32^. Each run included a negative (nuclease-free water) and a positive (field isolation) control.

##### Interpretation of real-time PCR

The Rotor-Gene Q thermal cycler (Qiagen®, Germany) was used for PCR amplification. The successfully amplified target gave an amplification curve and the cycle threshold (Ct) at which the target amplicon was initially recognized above the background fluorescence levels, according to SDS software. FMDV was then identified using baseline and graph-based threshold cycle (Ct) values. Amplification with Ct values of 32.0 was judged positive, whereas amplification with unknown Ct values was considered negative. Because the Ct values of 32 and 50 were equivocal, the test was repeated^37^. Positive responses resulted in a detectable Ct value^33^. Ct values for samples with strong positive FMD are less than 20.0^34^.

#### Serotype identification of FMD virus by antigen detection ELISA

The Foot and Mouth Disease Virus was serotyped using an antigen detection sandwich ELISA. Sandwich-ELISA was carried out with specific anti-FMDV monoclonal antibodies (MAb), both coated and conjugated. The kit was created to identify and categorize serotypes O, A, C, Asia-1, SAT-1, and SAT-2. To support FMDV serotyping, a pan-FMDV test was included in the kit, which identifies O, A, C, and Asia1 isolates. The test was conducted according to the manufacturer's instructions and OIE guidelines^23^.

Positive sample suspensions that tested positive for FMDV by molecular detection were required to be tested for serotype identification using sandwich ELISA on a 96-well microplate. After distributing 50 l of dilute buffer into all wells of the test plate, 50 l of previously diluted samples using ELISA buffer and ready-to-use controls were dispensed into the appropriate wells of the test plate precoated with recombinant FMD viral antibodies. Each plate comprised one positive control and one negative control for each FMD serotype of O, A, C, Asia-1, SAT-1, SAT-2, and pan O, A, C, and Asia1.

The plates were sealed using the included plate sealer and incubated for 1 h at room temperature (18–30 °C). The wells were emptied and violently tapped after incubation to remove any remaining residual fluids. The wells were then filled with 200 l of washing solution and incubated at room temperature for 3 min before being emptied and the washing cycle was repeated twice (a total of three washing cycles). All remaining fluids were removed using clean absorbent paper and 50 l of the conjugate. From rows A through F, the same volume of conjugate A was added, and from rows G and H, the same volume of conjugate B was added. Plates were covered and incubated at room temperature for an hour. Four washing cycles were conducted as specified above, with the final one lasting 5 min.

All wells received 50 l of substrate/chromogen solution, and plates were covered and left at room temperature for 20 min in the dark. Stopping the reaction was done by adding 50 l of the stop solution (sulfuric acid) in the same sequence as the substrate solution. The contents of the well had been mixed before reading. Immediately after stopping, the optical density (OD) of each well was measured at 450 nm using a microplate reader. The test validity and outcome criteria for the samples tested are interpreted as given in (Table 1).

**Table 1:** Interpretation of antigen detection ELISA result.

Antigen detection by ELISA	Interpretation
Negative For FMDV	OD ≤ 0.1
FMDV positive for type O	OD > 0.1 with the type O MAb and with the pan-FMDV MAb; some samples may cross-react with the 1st MAb type A, but OD values with MAB O are higher
FMDV positive for type A	OD > 0.1 with at least one of the two type A MAbs and with the pan-FMDV MAb
FMDV is positive for type Asia 1	OD > 0.1 with the type Asia 1MAb and with the pan-FMDV MAb
FMDV positive for type C	OD > 0.1 with the type C MAb and with the pan-FMDV MAb
FMDV positive for type SAT1	OD > 0.1 with the type SAT1 catching MAb; some samples could be positive also with the pan-FMDV MAb
FMDV positive for type SAT2	OD > with the type SAT2 catching MAb; some samples could be positive also with the pan-FMDV MAb
FMDV positive (untyped)	OD > 0.1 with the pan-FMDV catching MAb and ≤ 0.1 with the type-specific MAbs

OD values of the samples were interpreted by subtracting the OD value of each negative control from the OD value measured for the test sample with the corresponding coated MAb.

##### Criteria for the validity of antigen detection ELISA

The positive inactivated controls were expected to have OD values of 1.0 units or higher. In contrast, the negative controls for serotypes O, A, C, Asia 1, and Pan-FMDV were expected to have OD values of less than 0.1 units. The negative controls for serotypes SAT1 and SAT2 were expected to have OD values of less than 0.2 units.

### Data management and statistical analysis

The raw data was recorded and coded in a Microsoft Excel spreadsheet 2019, and analyzed by STATA software version 14. By dividing the number of seropositive samples by the total number of samples examined, the seroprevalence was computed. In logistic regression analysis, seroprevalence was utilized as an outcome variable against each of the explanatory variables of the hypothesized risk factors (breed, sex, age, body condition, herd size, grazing system, agroecology, and wildlife contact). Explanatory variables with a p-value <  = 0.25 (maximum likelihood ratio test) were chosen in univariable analysis for multiple logistic regression analyses. The final multiple logistic regression models were manually generated using a forward stepwise selection procedure. A variable was considered a confounder if it altered the coefficient of the significant variables by more than 25%. Kruskal gamma statistics were used to analyze the multicollinearity of the predictors in the models, and variables with gamma values between − 0.6 and + 0.6 were included in a multivariable logistic regression model. The odds ratios (OR) and 95% confidence intervals (CI) of the covariates connected to the outcome variables were calculated using the final multivariate logistic regression models. P-values less than 0.05 were used to determine significant differences.

### Ethical approval and consent to participate

The animal research ethics review committee of the Wolaita Sodo University Institutional Review Board has assessed the proposal and approved the work to be conducted and given an ethical certificate with reference number WSU 41/22/3230/2022. Blood samples were collected during routine veterinary practice in adherence to a high standard of veterinary care, and after the permission of the animal owners and informed consent was obtained from owners for animal use. The authors would confirm that manipulations on animals were conducted according to research animal guidelines and regulations of the School of Veterinary Medicine of Wolaita Sodo University which was in line with ARRIVE guidelines.

## Results

### Overall seroprevalence of FMD

There were 46.88% (180/384) positive serum antibodies against FMDV's non-structural protein in 384 blood samples tested. Seroprevalence was higher in Wolaita Sodo Town than in other districts (Table 2).

**Table 2:** Summary of the seroprevalence and risk factors in the study area.

Variables	Category	No. of examined	No. of positive	Prevalence (%)	[95% CI]
Breed	Local	308	144	46.75	41.22–52.37
	Crossbred	76	36	47.37	36.35–58.65
Sex	Male	210	105	50.00	43.24–56.75
	Female	174	75	43.10	35.90–50.61
Age	Adult	237	123	51.89	45.51–58.23
	Old	39	22	56.41	40.48–71.12
	Young	108	35	32.40	24.21–41.85
Herd size	Large	79	57	72.15	61.19–80.97
	Medium	102	61	59.80	49.95–68.91
	Small	203	62	30.54	24.56–37.25
Body condition scores	Good	232	113	48.71	42.29–55.15
	Medium	79	31	39.24	29.05–50.47
	Poor	73	36	49.32	37.96–60.73
Grazing system	Free	36	12	33.33	19.81–50.29
	Indoor	96	46	47.92	38.06–57.94
	Mixed	252	122	48.41	42.27–54.60
Agroecology	Highland	191	104	54.45	47.30–61.41
	Lowland	120	43	35.83	27.72–44.85
	Midland	73	33	45.21	34.12–56.78
Contact with wildlife	Yes	290	153	52.76	46.97–58.47
	No	94	27	28.72	20.43–38.73
Districts	Boloso sore	128	43	33.59	25.91–42.26
	Offa	128	56	43.75	35.37–52.50
	Sodo Town	128	81	63.28	54.55–71.22
Total		384	180	46.88	41.91–51.90

### Analyzing the association of risk factors with seroprevalence of FMD

The current study found that cross-breed, male, older age, larger herd size, poorly conditioned cattle, cattle grazing in a mixed grazing system, highland cattle, and cattle in contact with wildlife had higher seroprevalence (Table 2).

#### Animal-related risk factors of FMD seroprevalence

In the current study, the relationship between FMD seropositivity and intrinsic risk variables (age, sex, physical condition, and breed) was analyzed using univariable logistic regressions, and only age was found to be significantly linked with FMDV prevalence. FMD seroprevalence was significantly higher in older (56.41%) and adult (51.90%) animals than in young (32.41%). Adult cattle were 2.69 times more likely (95% CI 1.27–5.71; p = 0.009) to acquire FMD than young cattle. Male cattle had a greater seroprevalence of FMD (50%) than female cattle (43.1%). The difference in seroprevalence, however, was not statistically significant (p > 0.05) (Table 3).

**Table 3:** Univariable logistic regression analysis of risk factors with the seropositivity FMD.

Variables	Category	Prevalence (%)	COR	[95%CI]	p-value
Sex	Male	50.00	1.32	0.88–1.98	0.178
	Female	43.10	Ref	Ref	
Breed	Local	46.75	Ref	Ref	
	Cross	47.37	1.02	0.62–1.69	0.923
Body condition scores	Good	48.71	1.47	0.87–2.47	0.145
	Medium	39.24	Ref	Ref	
	Poor	49.32	1.50	0.79–2.86	0.212
Age	Adult	51.89	2.25	1.39–3.62	0.001
	Old	56.41	2.69	1.27–5.71	0.009
	Young	32.40	Ref	Ref	
Herd size	Large	72.15	5.89	3.31–10.47	≤ 0.001
	Medium	59.80	3.38	2.06–5.55	≤ 0.001
	Small	30.54	Ref	Ref	
Grazing system	Free	33.33	Ref	Ref	
	Indoor	47.92	1.84	0.82–4.09	0.135
	Mixed	48.41	1.87	0.89–3.92	0.093
Agroecology	Highland	54.45	2.14	1.33–3.42	0.001
	Lowland	35.83	Ref	Ref	
	Midland	45.21	1.47	0.81–2.67	0.197
Contact with wildlife	Yes	52.76	2.77	1.67–4.58	≤ 0.001
	No	28.72	Ref	Ref	

*COR* Crude odds ratio, *CI* confidence interval.

Crossbred cattle had a slightly higher frequency of FMD (47.37%) than indigenous breed cattle (46.75%). There was no statistically significant difference across breeds (p > 0.05). Furthermore, poorly conditioned cattle had a greater FMD seroprevalence (49.32%) than good-conditioned cattle, and there was no statistically significant variation in FMD seroprevalence and body condition score (Table 3).

#### Environment-related risk factors for FMD seroprevalence

Extrinsic risk factors for FMD occurrence included grazing systems (free, indoor, and mixed), herd size, contact with wildlife, and agroecology. There was an association between herd size, agroecology, animal contact, and FMD seroprevalence. However, FMD seroprevalence was high in mixed (48.41%) grazing systems, followed by indoor (47.92%) and it was statistically insignificant (p > 0.05).

In this study, animals in large herd numbers were 5.89 times more likely to acquire FMDV than those in medium herd sizes irrespective of other factors and it was statistically significant (p < 0.05). This could be a sign of the disease's infectious nature and mechanism of transmission, which is connected to herding size increasing animal crowding, which can allow the frequency of direct contact and so raise the possibility of FMD transmission.

The study found that the highlands had the highest FMD seroprevalence (54.45%), followed by the midlands (45.21%) and lowlands (35.83%), although there was no statistically significant relationship between agroecology and FMD occurrence (p > 0.05).

The seroprevalence of cattle in contact with wildlife (52.76%) and cattle having no contact with wildlife (28.72%), respectively, and cattle that have contact with wildlife are 4.89 times more likely (95% CI 2.70–8.88, p ≤ 0.001) to develop FMD as compared to cattle that have no contact with wildlife. This implies that the circulations of the wildlife (buffalo) in/near the study area play a great role in preserving the diseases besides the long-distance dissemination nature of the FMDV.

#### Multivariable logistic regression analysis of risk factors with FMD seropositivity

Only four of the eight hypothesized risk factors (herd size, body condition, age, and wildlife contact) showed a significant correlation and were included in the multivariate logistic regression model. The chance of becoming FMD seropositive was substantially greater in poorly conditioned cattle (49.32%; AOR = 2.50 (95% CI 1.21–5.24; p = 0.014)) than in good-conditioned cattle. The seroprevalence of FMD was substantially higher in large herd sizes [72.15%; AOR = 8.34 (95%CI 4.39–15.84, p ≤ 0.001)] than in medium herd sizes. Older cattle were found to be 3.27 times more seropositive [56.41%; 95% CI 41.39–7.69, p = 0.007] than young (Table 4).

**Table 4:** Multivariable logistic regression analysis of potential risk factors associated with the seropositivity FMD.

Variables	Category	Prevalence (%)	Multivariable logistic		
			AOR	[95%CI]	p-value
Age	Adult	51.89	3.20	1.84–5.58	≤ 0.001
	Old	56.41	3.27	1.39–7.69	0.007
	Young	32.40	Ref	Ref	
Herd size	Large	72.15	8.34	4.39–15.84	≤ 0.001
	Medium	59.80	4.42	2.54–7.69	≤ 0.001
	Small	30.54	Ref	Ref	
Contact with wildlife	Yes	52.76	4.89	2.70–8.88	≤ 0.001
	No	28.72	Ref	Ref	

*AOR* Adjusted Odds ratio, *CI* confidence interval.

### Molecular detection of FMDV from outbreak cases

**Figure 2:**
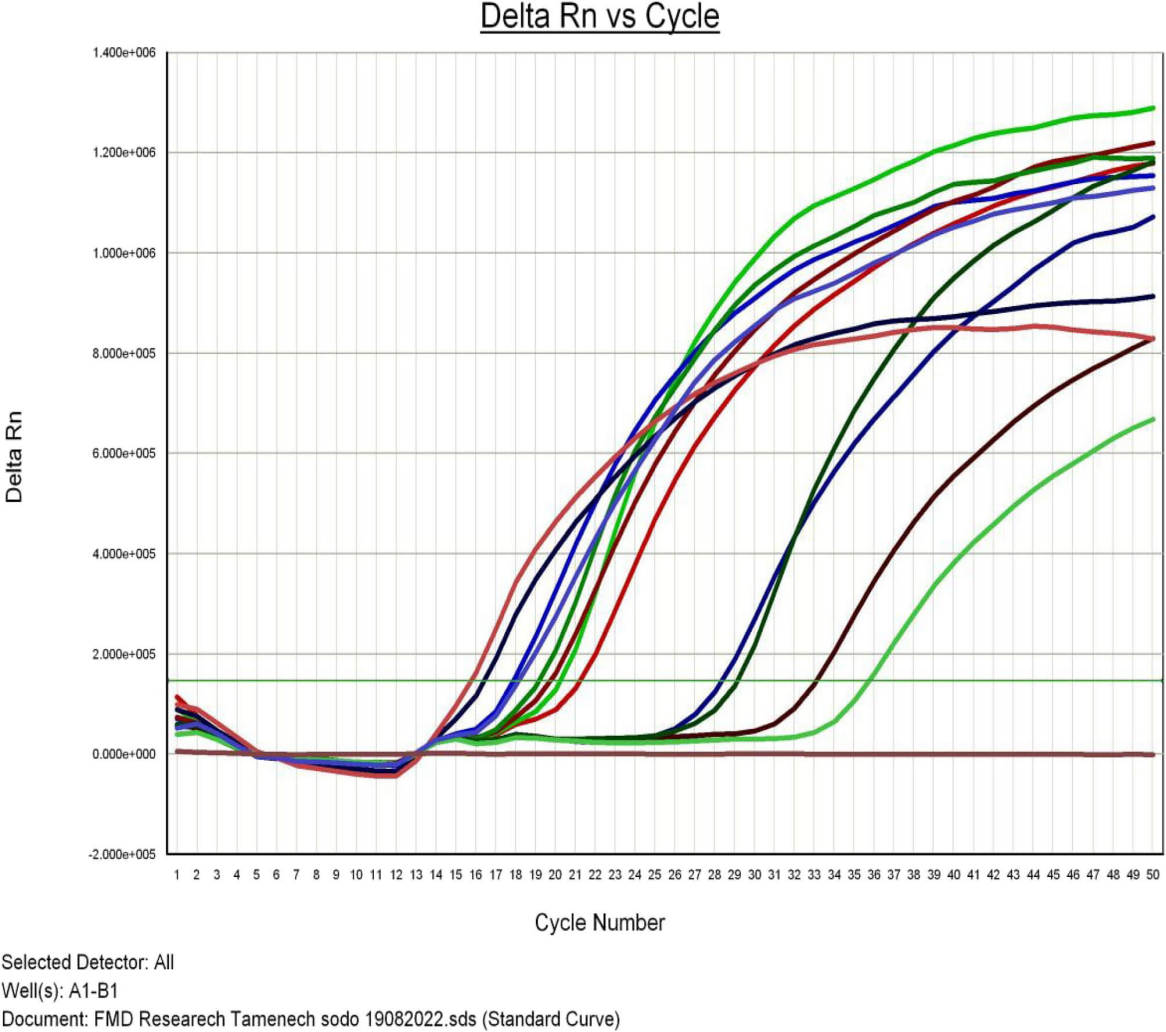
Real-time RT-PCR Positive results showing amplification curve (above threshold line).

The Ct value (cycle threshold or crossover point) is the number of cycles required for a given sample to exceed the above-mentioned threshold and is regarded as positive. Real-time RT-PCR (with universal primers and FMDV probe) was used to detect FMDV in all ten samples obtained. The Ct values for all ten samples ranged from 16.01 to 34.01, and the fluorescence of the samples rose above the background fluorescence (Fig. 2).

In the current study, real-time PCR was used to test 10 bovine epithelial tissue samples, and all were shown to be positive for FMDV 3D regions (Table 5). The presence of FMDV was confirmed in samples using real-time PCR, which yielded positive results above the threshold line, demonstrating that nucleic acid detection techniques are effective tools for rapid and sensitive disease diagnosis.

**Table 5:** Molecular detection and serotype identification of FMD virus from outbreak cases.

Sample code	Outbreak district	Sites of outbreaks	Sex	Age	Molecular detection	Serotyping results
BM1	Offa	Mancha	M	Adult	Positive	SAT-2
BF2	Offa	Mancha	F	young	Positive	SAT-2
BM3	Offa	Mancha	M	Adult	Positive	SAT-2
BM4	Offa	Mancha	M	Adult	Positive	SAT-2
BF5	Offa	Kodo	F	Adult	Positive	SAT-2
BF6	Offa	Kodo	F	young	Positive	SAT-2
BF7	Offa	Kodo	M	Adult	Positive	SAT-2
BF8	Offa	S/Esho	M	Adult	Positive	SAT-2
BM9	Offa	S/Esho	F	Adult	Positive	SAT-2
BM10	Offa	S/Esho	M	Adult	Positive	SAT-2

### Serotype identification of FMD Virus

FMD serotyping was performed on 10 samples using an antigen detection sandwich ELISA to determine the serotypes involved in the outbreaks. All ten outbreak samples that tested positive for FMDV in molecular detection were meant to identify circulating serotypes, and those outbreak-confirmed samples were identified as serotype SAT-2 (Table 5).

## Discussion

The current study found that 46.88% (95% CI 41.91–51.90) of the serum samples tested positive for FMDV's non-structural protein. The current study's findings were consistent with prior reports: 49.2% in Oromiya^35^, 41.5% in the eastern portion of Tigray Zone^36^, and 48.1% in southern Ethiopia^37^. However, there is a lower seroprevalence of FMD reported from previously conducted studies in different parts of Ethiopia; 8.9% in South Omo Zone^38^, and 21% in the Borana pastoral area, southern Ethiopia^10^.

On the contrary, a higher seroprevalence of FMD was reported at the country level with a prevalence of 53.6% in Ethiopia by Ayelet et al.^36^, 72.6% in Nigeria by Lazarus et al.^39^, and 72.1% by Awel et al.^22^ in Addis Ababa.

This might be attributed to variances in unrestricted animals’ movement^17,36^, livestock distribution, the amount of interaction between herds and wildlife, and grazing type in each administrative structure. Furthermore, vaccine matching is a significant difficulty in FMD immunization due to the presence of various serotypes and strains that do not cross-protect against each other^40^. Foot and mouth disease vaccines are frequently developed to cover many serotypes and strains, which reduces the efficacy and cost of the vaccinations when compared to monovalent vaccines^23^.

In the current study finding, the adult cattle were 2.69 times more (95% CI 1.27–5.71; p = 0.009) likely to have a chance of contracting FMD than young cattle. The current study finding was in line with previous study reports, which reported that adult cattle were 2.7 times more likely to contract the disease than young cattle in western Ethiopia, Bayissa et al.^41^ in Borna pastoral and agro-pastoral area, and Ishola et al.^42^, which reported a higher prevalence of FMD in adult cattle than in young ones.

On the other hand, reports by Rufael et al*.*^10^ in the Borana pastoral area; and Megersa et al.^37^ in Gamo Gofa and Sidama Zones; revealed significantly higher FMD seroprevalence in young as compared with adult and old cattle.

The age correlation with FMD seroprevalence might be attributed to increasing exposure to disease risk factors as an animal's age grows. Furthermore, young animals under 2 years old were frequently maintained separately throughout the homestead, resulting in a low frequency of viral exposure and protection against the disease from the predominant passive mother immunity^28^.

In the current study, animals in large herd numbers were 5.89 times more likely to acquire FMDV than those in medium herd sizes. There was also a statistically significant (p < 0.05) relationship between the disease and herd size, with rising herd size increasing seroprevalence of FMDV antibodies. This might be a sign of the disease's infectious nature and mechanism of transmission, which is connected to herding size increasing animal crowding, which can allow the frequency of direct contact and so raise the possibility of FMD transmission. The finding agreed with Bayissa et al.^41^, who reported that there was positive a relationship between FMD seroprevalence and herd size.

This study revealed that animals’ contact with wildlife was also considered as contributing risk factor for the occurrence of the disease in the study area and the seroprevalence of cattle in contact with wildlife and cattle having no contact with wildlife were 52.76% and 28.72%, respectively. Cattle that have contact with wildlife are 4.89 times more (95% CI 2.70–8.88, p ≤ 0.001) likely to develop FMD as compared to cattle that have no contact with wildlife. Similarly, the ungulate wildlife serves as a reservoir for the disease to circulate in the study area. This study finding agreed with previous studies by Molla and Delil^38^, in the South Omo zone who reported that cattle that regularly were in contact with ungulate wildlife were 3.3 times more likely to develop the disease than cattle having no contact with wildlife. The contact between wildlife and livestock at watering points and grazing areas is the main risk factor for FMDV circulation and it is a challenge for disease control in East Africa^39^.

In the present study, 10 bovine epithelial tissue samples were tested by real-time PCR, all were found positive for 3D regions of FMDV. Samples were confirmed for the presence of the FMDV by using real-time PCR that showed positive results above the threshold line, which explains that nucleic acid detection techniques are powerful tools for rapid and sensitive diagnosis of the disease. The results of real-time PCR agreed with Paixao et al.^43^ who reported that real-time RT-PCR that targets the 3D region of the viral genome is a powerful technique for reliable detection of FMDV which currently is becoming a key diagnostic test used to confirm FMDV presence in field samples.

The lower Ct values could indicate higher concentrations of the virus in the samples (Fig. 2). In support of this observation, OIE^23^ reported that the preferred sample for virus detection is the epithelial tissue which was previously confirmed by Urge et al.^35^ and Ayele et al.^36^ who reported the presence of higher levels of viral RNA in the epithelial tissues. In agreement with this, Reid et al.^29^ indicated epithelial tissue samples from the vesicular lesions could be used as the sample of choice for FMDV detection.

Ethiopia is one of the FMD-endemic countries in the Horn of Africa, with almost five serotypes prevailing so far. In Ethiopia, studies revealed that O, A, C, SAT-2, and SAT-1 serotypes were identified using serology and molecular techniques during the period 1981–2018 & and were responsible for FMD occurrence^10,31^. Moreover, antigen detection using sandwich ELISA revealed that the SAT-2 serotype was involved in the FMD outbreaks in the study districts. In support of this study’s findings, previous studies showed that serotype SAT 2 FMD viruses were identified from cattle found in Ludehitosa district (Arsi zone), Adama and Boset district (East Shewa zone), and Kolfe district (Addis Ababa)^14^.

Similarly, in many sub-Saharan African countries, Vosloo et al.^44^ suggest endemicity of the serotype in these countries. Furthermore, the International Organization for Animal Health (OIE) FMD disease occurrence report in Africa continent since 2000 to 2010 revealed that SAT-2 was escalating as an important serotype (41%) followed by O serotype (23%)^23^.

Moreover, Ayelet et al.^45^ reported isolation of SAT 2 in 1989–1991 from a cattle sample, and the virus was detected not again until 2007, an apparent gap of 16 years. Thus, up-to-date data on the SAT 2 serotypes was necessary as long as the an absence of cross-protection immunity between the FMDV vaccine. Also, the finding of this study tremendously helps to mitigate the impact of the circulating serotype on the livestock sector in vaccine manufacturing for existing serotypes in the study area.

As a limitation of the study, the vaccine efficacy test and serotype sequencing of FMD were not conducted.

## Conclusion

The current study found that FMDV is extremely common in cattle handled under various production techniques in the study region of selected districts in the Wolaita zone. Clinical examination, serological testing, and molecular detection techniques, particularly in the research region, demonstrated that serotype SAT-2 FMDV was circulating in the study area and was the cause of the disease outbreaks. The multivariable logistic regression revealed that age, body condition score, herd size, and interaction with wildlife all had a significant correlation with FMD seropositivity risk variables.

In conclusion, the regional government should emphasize massive vaccination campaigns, and create awareness through training of smallholder farmers. The restriction of free movement of livestock with neighboring zones and the establishment of quarantine stations around the border area was supported.

## Data Availability

This manuscript includes all the datasets generated or analyzed during this study.

## Abbreviations

Ct: Cycle threshold
DIVA: Differentiating Infected from Vaccinated animals
DNA: Deoxyribonucleic acid
RNA: Ribonucleic acid
FMD: Foot and mouth disease
FMDV: Foot and mouth disease virus
ELISA: Enzyme-linked immunosorbent assay
NAHDIC: National Animal Health Diagnostic and Investigation center
NSP: Non-Structural Protein
OD: Optical density
OIE: Office International des Epizooties
SAT: South African Territories
PCR: Polymerase chain Reaction
RT-PCR: Reverse transcription Polymerase chain Reaction
rRT-PCR: Real-time Reverse transcription Polymerase chain Reaction
